# Association of Opium Consumption and Coronary Artery Ectasia: A Propensity Score-Matched Study

**DOI:** 10.34172/aim.2022.86

**Published:** 2022-08-01

**Authors:** Mahan Shafie, Arya Aminorroaya, Ali Vasheghani-Farahani, Arash Jalali, Abdolvahab Baradaran

**Affiliations:** ^1^School of Medicine, Tehran University of Medical Sciences, Tehran, Iran; ^2^Tehran Heart Center, Tehran University of Medical Sciences, Tehran, Iran; ^3^Non-Communicable Diseases Research Center, Endocrinology and Metabolism Population Sciences Institute, Tehran University of Medical Sciences, Tehran, Iran; ^4^Cardiac Primary Prevention Research Center, Cardiovascular Diseases Research Institute, Tehran University of Medical Sciences, Tehran, Iran

**Keywords:** Coronary aneurysm, Coronary angiography, Coronary artery ectasia, Markis classification, Opium

## Abstract

**Background:**

Opium consumption is associated with increased risk of atherosclerosis and a hyper-inflammatory state which are suggested as contributing factors to the development of coronary artery ectasia (CAE). We aimed to determine if opium consumption is an independent risk factor of CAE. This study aimed to explore the relationship between opium consumption and CAE.

**Methods:**

In this propensity score-matched study, we enrolled patients who underwent elective coronary angiography between September 2004 and March 2017 in Tehran Heart Center. We studied patients with CAE and without coronary artery disease (CAD) as cases. The control group, patients with normal coronary angiograms, were selected after applying the propensity score matching to match for age, sex, diabetes mellitus, hypertension, hyperlipidemia, family history of coronary artery disease, and cigarette smoking.

**Results:**

We studied 242 patients with pure CAE and selected 968 control patients. The prevalence of opium consumption was not significantly different across these groups: 17 (7.5%) in the pure CAE group compared to 76 (8.6%) in the control group (Odds ratio: 0.81; *P*=0.455). Amongst the patients with pure CAE, Markis scores were not significantly different between opium consumers and non-consumers (*P*=0.136).

**Conclusion:**

We found no significant difference regarding opium consumption between patients with pure CAE and those with normal coronary angiograms. In addition, there was no correlation between opium consumption and Markis scores in patients with pure CAE.

## Introduction

 According to the World Drug Report 2020, opioids, including opiates and synthetic opioids, are the second most commonly used drugs after cannabis globally; however, they are the most harmful drugs in terms of morbidity and mortality. In 2017, opioids were responsible for about 66% of the mortality and 80% of the disability-adjusted life years attributed to drug use disorders worldwide. The prevalence of opioid use has increased recently and it is estimated that about 57.8 million 15–64-year-old people use opium or other opioids around the world in 2018. In this year, the prevalence of past-year use of opiates including opium and heroin was greater than the global average in the Middle East, and South-West and South Asia in which almost one-third of the world opiate users reside.^1^ Opium is the most commonly abused substance in many Asian and Middle Eastern countries after cigarette smoking. This fact may be attributed to its greater availability in these regions which might arise from their proximity to the production sources of opium like Afghanistan.^2,3^ Another reason may be a false traditional belief that opium consumption ameliorates cardiovascular diseases (CVD) and cardiovascular risk factors.^3-5^

 Studies have demonstrated the association between opium and CVD and underscored the importance of opium-induced inflammation in this regard.^3^ Although some studies have reported neutral or even beneficial effects of opium consumption on cardiovascular health, many investigators found its detrimental effects on CVD and atherosclerosis.^3,6,7^ A recent systematic review and meta-analysis demonstrated that opium use is associated with a 2.75-fold increased risk of coronary artery disease (CAD) (odds ratio: 2.75; 95% confidence interval: 2.04 to 3.75).^7^ In addition, studies report that opium use is associated with chronic inflammation and oxidative stress. Opium may cause increased levels of reactive oxygen spices and other inflammatory components. Moreover, it is associated with decreased antioxidant capacity and suppressed anti-inflammatory responses.^3,7^ Hence, opium consumption is interwoven with CVD and inflammation.

 Coronary artery ectasia (CAE) is the dilatation of a portion of a coronary artery with a prevalence of 1.2%–4.9% on coronary angiograms.^8^ Studies demonstrate that CAE, similar to CAD, is a variant of atherosclerosis and its diagnosis bears important prognostic implications comparable with CAD in terms of mortality.^8-11^ To date, the exact pathogenesis of CAE is not fully defined. In addition to atherosclerosis, congenital anomalies, and iatrogenesis, studies have suggested inflammation as a contributing factor to CAE.^12-14^

 Investigators have found that opium consumption is associated with increased risk of atherosclerosis and a hyper-inflammatory state,^3,6,7^ which have been suggested as contributing factors to the development of CAE; however, few studies have examined the possible association between opium use and CAE.^15^ In this study, we aimed to evaluate this association and determine if opium consumption is an independent risk factor of CAE.

## Materials and Methods

###  Design and Participants

 We reported this study according to the STrengthening the Reporting of OBservational studies in Epidemiology (STROBE) statement.^16^ In this propensity score-matched study, we enrolled patients who underwent elective coronary angiography (CAG) between September 2004 and March 2017 in Tehran Heart Center in Tehran, Iran.^17^ We considered all patients aged at least 18 years who underwent elective CAG. We studied patients with CAE and without CAD as cases. Patients with normal CAG were designated as the control group. Patients with congenital or valvular heart disease, autoimmune diseases including scleroderma, systemic lupus erythematous and Behcet’s disease, or connective tissue disorders including Marfan syndrome and Ehlers-Danlos syndrome were excluded.

 We retrieved all of the required data of the patients from their electronic medical records using Tehran Heart Center’s data registry. This data registry comprises demographic, procedural, and post-procedural data which are collected, reviewed, and fed into relevant data bank systems by the treating physicians or trained general practitioners/research nurses. Thereafter, the data are refined and cleaned by experts as part of quality control.^17^

 Based on the Tehran Heart Center’s angiography database, the frequency of opium consumption among control participants who showed normal result of CAG was about 9%. Considering previous studies to detect an odds ratio of at least 1.835, given a significance criterion of 0.05 and 80% power, a sample size of 242 CAE cases and 968 controls would be required for this study.

###  Definitions

 CAE is the dilatation of a portion of a coronary artery 1.5 times greater than the diameter of the adjacent normal coronary artery.^18^ We categorized CAE according to the Markis classification into four groups: (1) Diffuse ectasia of two or three vessels; (2) Diffuse ectasia in one vessel and localized ectasia in another vessel; (3) Diffuse ectasia in one vessel only; (4) Localized or segmental involvement.^19^ All CAGs were reviewed by attending cardiologists promptly after the procedure to determine the diagnosis. In this study, another expert cardiologist (AB) reviewed the CAGs with the diagnosis of CAE to confirm the diagnosis and determine Markis scores.

 We defined hyperlipidemia as total cholesterol ≥ 200 mg/dL, or high-density lipoprotein (HDL) ≤ 40 mg/dL in men and ≤ 50 mg/dL in women, or low-density lipoprotein (LDL) ≥ 100 mg/dL, or triglycerides ≥ 150 mg/dL, or being on lipid-lowering agents. Hypertension was designated as systolic blood pressure ≥ 140 mm Hg, or diastolic blood pressure ≥ 90 mmHg, or receiving antihypertensive medications. Fasting blood sugar (FBS) ≥ 126 mg/dL on two occasions, or 2-hour postprandial blood sugar ≥ 200 mg/dL on two occasions, or random blood sugar ≥ 200 mg/dL accompanied by symptoms of diabetes mellitus (DM), or receiving either oral antidiabetic agents or insulin were defined as DM. Opium users were the ones who consume opium regularly either by inhalation or ingestion, and opium non-users were the patients who have never used opium.

###  Statistical Analysis

 We described continuous variables as mean with standard deviation for normally distributed variables and median [interquartile range boundaries (IQR)] for non-normally distributed variables. Continuous variables with normal distribution were compared using independent samples *t*-test and we used the Mann-Whitney U test to compare non-normally distributed variables. Categorical variables were presented as number (percentage) and compared by the Chi-square test. Due to ordinal nature of the Markis score, it was compared between opium consumer and non-consumer groups applying a Mann-Whitney U-test. Moreover, a Fisher’s exact test was used to compare the frequency distribution of Markis scores between the above-mentioned groups. We employed propensity score matching (PSM), a statistical approach which attempts to ensure balance between pure CAE and normal CAG groups, by matching groups for possible confounders. We matched pure CAE patients with a ratio of 1:4 using the nearest neighbor technique on the propensity scores derived from a logistic regression model of pure CAE on age, sex, DM, hypertension, hyperlipidemia, family history of CAD, and cigarette smoking. A conditional logistic regression model was applied to assess the association of opium consumption and pure CAE, and the estimated effect was reported as odds ratio (OR) with 95% confidence interval (CI). Data analyses were done using IBM SPSS Statistics for Windows, version 25.0 (Armonk, NY: IBM Corp.)

## Results

 We evaluated the results of 119 218 elective CAGs from September 2004 to March 2017 in Tehran Heart Center. Of these 119 218 CAGs, there were only 242 patients with CAE and without CAD designated as pure CAE. Therefore, we included 242 pure CAE patients and selected 968 individuals from 16,522 patients with normal CAGs as the control group using PSM. There was no data regarding opium consumption in 15 (6.2%) patients with pure CAE and 80 (8.3%) patients with normal CAG. According to the implementation of the PSM, baseline characteristics were not statistically different across these groups except for higher levels of plasma LDL, total cholesterol, and triglyceride in patients with normal CAG compared to patients with pure CAE (Table 1).

**Table 1:** Baseline Characteristics of Patients after Propensity Score Matching

Characteristic*	**Normal CAG (n=968)**	**Pure CAE (n=242)**	***P *** **Value**
Age (y)	54.88 ± 11.43	54.93 ± 10.95	0.947
Gender (male)	559 (57.7%)	138 (57.0%)	0.839
DM	119 (12.3%)	27 (11.2%)	0.627
Hypertension	437 (45.2%)	116 (47.9%)	0.452
Hyperlipidemia	543 (56.3%)	131 (54.6%)	0.638
Family history of CAD	139 (14.5%)	30 (12.4%)	0.418
Cigarette smoking	136 (14.0%)	38 (15.7%)	0.512
Opium use	76 (8.6%)	17 (7.5%)	0.603
BMI (kg/m^2^)	29.62 ± 5.59	29.62 ± 5.75	0.987
FBS (mg/dL)	96.00 [89.00–107.00]	97.00 [89.00–107.00]	0.908
HDL cholesterol (mg/dL)	43.20 ± 11.61	43.91 ± 12.01	0.442
LDL cholesterol (mg/dL)	104.00 [81.00–129.00]	99.50 [75.00–122.50]	0.044
Triglyceride (mg/dL)	136.00 [99.00–186.00]	122.00 [90.00–168.00]	0.007
Total cholesterol (mg/dL)	172.00 [147.00–205.00]	165.00 [137.00–197.00]	0.011

*Note*. Data are presented asmean ± standard deviation, median [percentile 25-percentile 75], or number (%). BMI, Body mass index; CAE, Coronary artery ectasia; CAD, Coronary artery disease; CAG, Coronary angiography; DM, Diabetes mellitus; FBS, Fasting blood sugar; HDL, High-density lipoprotein; LDL, Low-density lipoprotein.

 After employing PSM and controlling for the effects of potential confounders, the prevalence of opium consumption was 17 (7.5%) in the pure CAE group versus 76 (8.6%) in the normal CAG group which was not significant after applying a conditional logistic regression (OR: 0.81, 95% CI: 0.46 to 1.41; *P* = 0.455). Among patients with pure CAE, 17 patients were opium users who had a median Markis score of 1.5 (IQR boundaries: 1–3), and 210 patients were opium non-user with a median Markis score of 2 (IQR boundaries: 1–4), which showed no significant difference (*P* = 0.136). The Markis classes were also not significantly different between these groups (*P* = 0.480) (Table 2).

**Table 2:** Markis Score in Patients with Pure Coronary Artery Ectasia

		**Opium Consumer** ^a^ ** (n=17)**	**Opium Non-Consumer** ^a^ ** (n=210)**	***P *** **Value**
Markis category	Class 1	7 (41.2%)	68 (32.4%)	0.480
	Class 2	3 (17.6%)	33 (15.7%)	
	Class 3	2 (11.8%)	32 (15.2%)	
	Class 4	1 (11.8%)	64 (30.5%)	
Markis score		1.5 [1-3]	2 [1–4]	0.136

*Note*. Data are presented as number (%), median [percentile 25-percentile 75]. ^a^There was no data regarding CAG in 3 (17.6%) opium consumers and 13 (6.2%) opium non-consumers.

Table 3 demonstrates the comparison of baseline characteristics between opium consumers versus non-consumers. Opium consumers were more likely to be younger, male, and smoke cigarettes, while they were less likely to have hypertension and hyperlipidemia compared to non-consumers. Furthermore, the body mass index (BMI), and the plasma level of HDL and total cholesterol were significantly lower in opium consumers compared to non-consumers.

**Table 3:** Comparison of Baseline Characteristics between Opium Consumers vs Non-consumers

Characteristic*	**Opium Consumer (n=93)**	**Opium Non-consumer (n=1022)**	***P *** **Value**
Age (years)	53.04 ± 9.06	55.25 ± 11.45	0.030
Gender (male)	90 (96.8%)	551 (53.9%)	< 0.001
DM	9 (9.7%)	127 (12.4%)	0.438
Hypertension	29 (31.5%)	483 (47.3%)	0.004
Hyperlipidemia	39 (41.9%)	579 (56.9%)	0.005
Family history of CAD	8 (8.6%)	147 (14.5%)	0.118
Cigarette smoking	54 (58.1%)	107 (10.5%)	< 0.001
BMI (kg/m^2^)	27.72 ± 5.85	29.85 ± 5.61	0.001
FBS (mg/dL)	95.50 [90.00–104.50]	97.00 [90.00–108.00]	0.740
HDL cholesterol (mg/dL)	40.72 ± 9.56	43.50 ± 11.98	0.016
LDL cholesterol (mg/dL)	98.00 [81.00–115.00]	104.00 [80.00–128.00]	0.242
Triglyceride (mg/dL)	121.00 [89.00–168.00]	130.50 [97.00–180.00]	0.238
Total cholesterol (mg/dL)	161.50 [138.00–188.00]	170.00 [146.00–203.00]	0.015

* Data are presented asmean ± standard deviation, median [percentile 25-percentile 75], or number (%). BMI, Body mass index; CAE, Coronary artery ectasia; CAD, Coronary artery disease; CAG, Coronary angiography; DM, Diabetes mellitus; FBS, Fasting blood sugar; HDL, High-density lipoprotein; LDL, Low-density lipoprotein.

## Discussion

 This study aimed to explore the relationship between opium consumption and CAE. We selected patients with CAE and without any co-existing CAD as our cases because about 50% of CAE is attributed to concomitant CAD in adult patients with CAE.^20^ Moreover, we employed PSM to consider the possible confounding effects of traditional cardiovascular risk factors as indicators of atherosclerosis. After taking these considerations into account, we found that the frequency of opium consumption was not significantly different between patients with pure CAE and patients with normal CAG.

 In a recent study, Bahrami et al reported that opium is an important risk factor for CAE. In their cross-sectional study, they enrolled 46 patients with CAE, 30 patients with CAD, and 42 cases without CAE and CAD. That study revealed that opium consumption was significantly greater in patients with CAE and CAD compared to controls. The number of participants in their study was much smaller than ours and it can be one of the reasons to explain the different results. Moreover, in that study, they did not adjust for other CVD risk factors including age, sex, DM, hypertension, hyperlipidemia, family history of CAD, and cigarette smoking between cases and controls while we used PSM to control the effect of any confounders and it could be another reason to explain the difference.^15^

 The role of opium consumption in the pathogenesis of CVD and the mechanisms involved in increasing the risk of CVD are not yet fully defined. Some studies suggest that inflammation caused by opium may play the main role in developing CVD, especially atherosclerosis, but whether this factor can cause CAE without any co-existing stenosis by itself has not yet been considered.^3,7^ In this study, we found that opium consumers were more likely to be male and smoke cigarettes, and had lower plasma levels of HDL cholesterol that make them more susceptible to CVD; nevertheless, they were younger, had lower plasma levels of total cholesterol, and were less likely to have hypertension or hyperlipidemia which are cardioprotective. We may hypothesize that these contradictive factors eventually resulted in no significant association between opium consumption and CAE. It is noteworthy that due to using PSM to match pure CAE and normal CAG groups, these results are not representative of our database and they can only describe the characteristics of the patients included in this study.

 Hyperlipidemia is another possible mechanism that can be induced by opium to develop CVD. Some studies have shown that opium may affect the level of HDL, LDL, total cholesterol, and triglyceride and with these effects, it may play a role in developing CVD.^21^ In addition, the association between CAE and traditional cardiovascular risk factors is still controversial but some studies have reported that CAE appears to be associated with these risk factors including hyperlipidemia.^22^ Although we used PSM to match baseline characteristics regarding hyperlipidemia, the levels of LDL, triglyceride, and total cholesterol were lower among CAE patients in comparison to normal CAGs (Table 1). Despite these statistical differences, there may not be a clinically significant difference. It should be noted that the lower levels of these three in patients with CAE could impact the relationship between opium and CAE in our study.

 To the best of our knowledge, this is one of the first studies dedicated to evaluating the association between opium use and CAE. We tried to conduct it as methodologically soundly as we were able; nevertheless, it has several limitations. First, it is an observational study that bears some inherent biases and falls short in establishing the causal relationship. Second, the missing data on opium consumption might result in bias in our findings. Due to the rarity of pure CAE, we acknowledge that the small number of pure CAE in our database would certainly be one of our limitations. Accordingly, future prospective cohort studies with larger sample sizes will address these limitations. Finally, the amount, frequency, and duration of opium consumption and cigarette smoking could have had an impact on the results of the study which did not quantify in the present study due to database limitations and this could be an issue to investigate in further studies.

 In conclusion,in this propensity score-matched study, we found that there was no significant difference regarding opium consumption between patients with pure CAE and patients with normal CAG. Additionally, there was no correlation between opium consumption and Markis category in patients with pure CAE. Future studies are needed to determine the exact role of opium consumption in CAE.
